# 129. Evaluation of Two-Step *Clostridioides difficile* Testing on Treatment Exposure

**DOI:** 10.1093/ofid/ofad500.202

**Published:** 2023-11-27

**Authors:** Nicholas Bennett, Sydney Wilson, Laura Aragon, Sarah E Boyd

**Affiliations:** Saint Luke's Health System, Kansas City, Missouri; Saint Luke's Hospital of Kansas City, Kansas City, Missouri; Saint Luke's Health System, Kansas City, Missouri; Saint Luke's Health System, Kansas City, Missouri

## Abstract

**Background:**

*Clostridioides difficile* can colonize or cause symptomatic disease from mild diarrhea to severe colonic inflammation requiring surgical intervention. Multiple *C. difficile* diagnostics are available and national guidelines recommend standalone or combination testing methods. Testing has mostly shifted to polymerase chain reaction (PCR)-based, which has shown up to a 50% increase in reporting of *C. difficile* infection (CDI). A two-step testing method has also been used to discern colonization versus active infection. The National Healthcare Safety Network has proposed changes to CDI reporting to include results and treatment, emphasizing the importance of diagnostic and antimicrobial stewardship.

**Methods:**

In 2021 Saint Luke’s Health System moved from *C. difficile* PCR standalone to two-step testing with PCR and enzyme immunoassay (EIA) toxin test. If PCR is positive, an EIA is run. Both results are reported including interpretation guidance. A retrospective cohort study was conducted to evaluate if two-step testing impacts antibiotic prescribing rates for *C. difficile*. The pre-implementation group included PCR-only and post- implementation was split into PCR +/ EIA - and PCR +/ EIA + groups.

**Results:**

Treatment rates for *C. difficile* did not significantly change after implementation of two-step testing (100% pre- vs 92% PCR +/ EIA - and 97% PCR +/ EIA +, p = 0.093). For PCR +/ EIA - results, treatment initiation more often occurred prior to results being available compared to the post-implementation PCR +/ EIA + group and pre-implementation group (30.3% PCR +/ EIA - vs 19.6% PCR +/ EIA + and 7.8% pre-implementation, (p = 0.028)). *C. difficile* antibiotic prescribing rates at discharge were no different between groups as the highest rates of treatment continuation were seen in the PCR +/ EIA - group (79.4% PCR +/ EIA - 72.5% pre-implementation vs 71.7% PCR+/ EIA +, p = 0.706).

**Conclusion:**

Despite PCR +/ EIA - results, clinicians tended to treat patients irrespective of PCR-only or two-step results. The impact of two-step *C. difficile* testing on treatment rates was limited and prescribing rates did not significantly change. The findings of this study warrant investigation into alternative solutions to promote aligned diagnostic and antimicrobial stewardship.

**Disclosures:**

**All Authors**: No reported disclosures

